# Cell lineage analysis of the mandibular segment of the amphipod *Orchestia cavimana *reveals that the crustacean paragnaths are sternal outgrowths and not limbs

**DOI:** 10.1186/1742-9994-3-19

**Published:** 2006-12-04

**Authors:** Carsten Wolff, Gerhard Scholtz

**Affiliations:** 1 Humboldt-Universität zu Berlin, Institut für Biologie/Vergleichende Zoologie, Philippstr. 13, 10115 Berlin, Germany

## Abstract

The question of arthropod head segmentation has become one of the central issues in Evolutionary Developmental Biology. The number of theories pertaining to head segments progressively enlarges, old concepts have been revitalized, and nearly every conceivable composition of the arthropod head has at some point received discussion. One contentious issue involves a characteristic mouthpart in crustaceans – the lower lips or the so-called paragnaths. The paragnaths build the posterior border of the mouth region antagonistic to the upper lip – the labrum. We show here the development of the appendage-like structures in the mandibular region of the amphipod crustacean *Orchestia cavimana *at a high level of cellular resolution. The embryos are examined during development of the mouthparts using *in vivo *labeling. An invariant cell division pattern of the mandibular segment was detected by 4D-microscopy and a preliminary model for pattern of the first cleavages in the mandibular region created. With this indispensable precondition single ectodermal cells of the grid-like pattern were labeled with DiI – a lipophilic fluorescent dye – to trace cell lineages and determine the clonal composition of the developing mouthparts, especially the mandibular segment. From our data it is evident that the paragnaths are sternal outgrowths of the mandible segment. The assumption of the limb nature of paragnaths and the presence of an additional head segment between the mandibular and the second antennal segments are clearly refuted by our data. Our results show the power of cell lineage and clonal analyses for inferences on the nature, origin and thus homology of morphological structures. With this kind of investigation morphological and gene expression data can be complemented.

We discuss notable similarities of paragnath anlagen to those of the hypopharynx complex in myriapods and hexapods. The fact that both structures grow out as two lateral buds in the same region of the mandibular sternite during development, and their important role in the formation of the feeding apparatus as a highly specialized chewing chamber in adults of crustaceans, myriapods, and hexapods argue for the paragnaths/hypopharynx anlagen being an additional potential apomorphy of Mandibulata.

## Background

**Figure 1:**
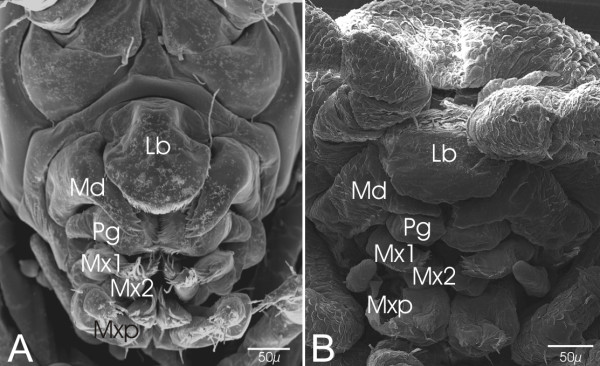
(**A**-**B**) Examples of shape and position of the crustacean paragnaths. (**A**) SEM-picture of the mouthparts of a hatchling of *Orchestia cavimana*. The incisor part of the mandibles (Md) covers partially the paragnaths. (**B**) SEM-picture of the mouthparts of a hatchling of the woodlouse *Porcellio scaber*. (Lb: Labrum; Md: Mandible; Mx1: Maxilla1; Mx2: Maxilla2; Mxp: Maxilliped)

The number and nature of the metameric elements constituting the head is one of the highly controversial issues in developmental biology, morphology, and phylogenetics of arthropods [1,2]. The most controversial head structure is the arthropod labrum, or upper lip. Based on a great variety of approaches and evidence the labrum is interpreted as being a derived pair of limbs with unclear segmental affiliations, a simple outgrowth, an anterior segment or the anterior most non-segmental body terminus, the acron [1,3]. Another structure of unresolved nature is the paired lower lip which is called paragnaths in crustaceans or superlinguae/hypopharynx in hexapods and some myriapods [4,5]. Paragnaths have been described for malacostracans, branchiopods, copepods, ostracods, mystacocarids, and cephalocarids (e.g. Ref. [4,6-10]). They are located in the posterior area of the mandibles and form often the lower border of the pre-oral cavity [4,11,12] (Fig. 1). Paragnaths play a role in feeding and in some taxa they are movable and have a complex and appendage-like appearance [13]. Moreover, in many crustaceans they are embryologically formed as limb-bud like outgrowths (e.g. Ref. [14-18]).

The superlinguae of many hexapods and Pauropoda, Symphyla and Diplopoda among the myriapods form, together with the unpaired lingua, the hypopharynx, a tongue-like structure at the posterior of the pre-oral cavity [5,19-22]. As is the case for the crustacean paragnaths, the hypopharynx/superlinguae are involved in food processing and embryologically they are formed as two processes at the posterior stomodeal region [23-25].

Although these paragnathal/superlingual/hypopharyngeal structures are not so much a focus of the general head debate as is the labrum, they present a similar set of problems. First, it is not clear whether paragnaths and superlinguae are homologous (see Ref. [5,26,27]). Second, based on early development, the sometimes complex adult structure, and the innervation pattern some authors interpret the paragnaths as being derived from limbs (e.g. Ref. [26,28,29]) or as parts of limbs [12,30,31] whereas others dispute this [10,32]. Third, the segmental relation is seen controversially either as postoral lip not related to any particular segment [7], as part of the mandibular segment [10,33], the segment of the first maxillae [8,12], or even the second maxillae [9]. Hansen [28], Denis [34], Chaudonneret[26], Laverack [35], and Casanova [29] suggest that the paragnaths indicate an additional segment either between mandibular and maxillary segments [28] or the tritocerebral and mandibular segments [26,29,34,35], concluding that the arthropod or mandibulate head comprises one more segment than generally thought. Lauterbach [32] hypothesized the origin of the paragnaths in sternal folds ("sternale Falten") of ancestral arthropods. According to Lauterbach the paragnaths are the result of progressive bulging and fusion of sternal elements of the first post-oral head segments in the Mandibulata, though only in some crustacean taxa do these folded sternal formations ("Faltenbildungen") have an appendage-like appearance.

**Figure 2:**
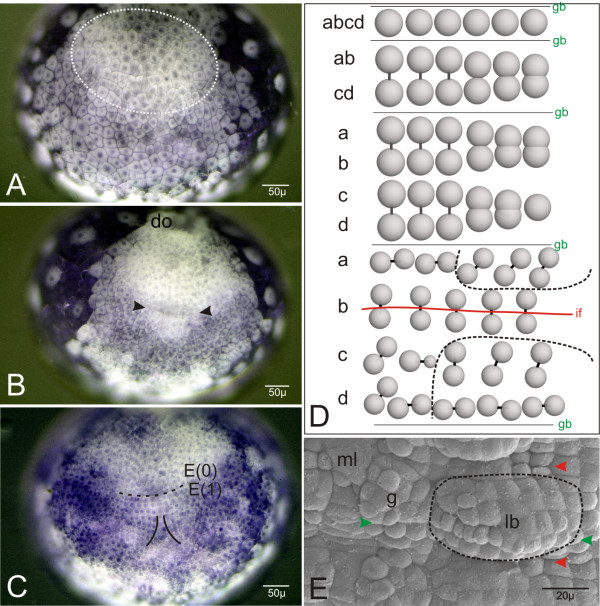
(**A**-**C**) Ventral view of living embryos of *Orchestia cavimana*. Anterior is up. (**A**) A typical germ disc stage after closing blastopore with around 250 cells. The dotted line shows the multi-layered region after gastrulation. (**B**) The embryo in **A **slightly more developed. The germ disc has now about 400 cells and a dorsal organ (do) is differentiated. In the centre of the germ disc the first formation of transverse rows is visible (black arrows). (**C**) The embryo in **A **further developed. The anterior lying dorsal organ is out of focus. The first transverse rows, beginning with E(1), are arranged in the typical grid like pattern. The dotted line indicates the border between the naupliar (posterior end marked by the genealogical unit E(0)) and the post-naupliar region (anterior end indicated by genealogical unit E(1)). (**D**) Schematic cell division pattern of post-naupliar ectoderm rows posterior to E(1) of *Orchestia *as is similarly found in other malacostracan crustaceans. Top: Beginning with the segment of the first maxilla, a non-teloblastic arranged genealogical unit (row **abcd**) undergoes two mitotic cleavage waves in a longitudinal direction. Middle: The result is a grid like pattern of four rows (from anterior to posterior: **a**, **b**, **c **and **d**). Bottom: After the first differential cleavages (bars mark sister cells) the first morphogenesis takes place. Interestingly the later morphological segments do not conform to this genealogical unit. The segmental border (if) runs between the descendants of row **b **and does not match the genealogical border (gb). Accordingly, limb buds, ganglia, and other segmental organs are composed structures formed by the descendants of two adjacent ectoderm rows. The dotted lines indicate the area of the later limb buds. The anteriormost post-naupliar ectoderm row E(1) shows a somewhat different pattern (see Fig. 4 and Ref. [36]). (**E**) SEM-picture of an early limb bud (surrounded by dotted line) of the left second thoracopod (first pereiopod). According to the color code in 1D the red arrowheads mark the intersegmental furrows and the green arrowhead marks the border of two consecutive genealogical segments which together form a morphological segment. Medial to the limb bud (lb) the anlage of the ganglion (g) of this hemi-segment and the slightly sunken midline (ml) is visible.

Here we address the problem of the nature and origin of crustacean paragnaths with a cell lineage approach. To resolve the segmental affinities of the paragnaths we study the cell lineage in the area around the mandibular segment of the freshwater beach-hopper, the amphipod crustacean *Orchestia cavimana*. This animal is well suited for this kind of investigation because it forms a pair of large buds of paragnaths during embryonic development [17]. Furthermore, as an amphipod representative its stereotypic cell division pattern in the post-naupliar region (segments of the first maxillae to the terminal segment of the pleon) is known (for this see Fig. 2D) and has been described up to the formation of morphological structures such as limbs, segments, and ganglia (Fig. 2E) [36]. In contrast to the well studied post-naupliar region, the processes of cell division and morphogenesis of the segments of the first and second antennae and the mandible (i.e. the naupliar region) are less known. This is due to the more irregular mode of cell division which, apart from some indications in the posterior mandibular region, does not show an obvious stereotypic pattern [36,37].

We combine the methods of 4D-microscopy [38] and the in-vivo labeling of single cells with the fluorescent dye DiI [39] to resolve the cell division pattern in the posterior naupliar region to trace the origin and formation of the paragnaths and other mouthparts by analyzing the clonal composition of the mandibular segment and adjacent areas.

It can be shown that the posterior region of the mandibular segment shows an unexpected degree of cell division determination with a reproducible cell lineage. The clear-cut results of our study shed new light on the segmentation pattern of crustacean heads by dismissing some older hypotheses on the origin and nature of paragnaths. The comparison and discussion of putative homologous structures in other arthropod taxa offer new perspectives on arthropod heads in general.

## Methods

### Culture

Specimens of the semi-terrestrial amphipod species *Orchestia cavimana *were collected from beaches of the Tegeler See (Berlin). The animals were maintained in a terrarium at 18–20°C and fed with carrots and oatmeal. To receive eggs in relevant stages gravid females were caught and isolated. The egg-bearing females where carefully anaesthetized in mineral water containing CO_2_. In their ventral brood pouch – the marsupium – the purple colored eggs are easy to recognize. The eggs were flushed out with a Pasteur pipette and transferred to a saline solution that mimics the osmotic milieu in the marsupium (details described in Wolff and Scholtz [39]).

### 4-D-micoscropy

Embryos in relevant stages (shortly before the first ectodermal rows appear) were mounted on microscopic slides. A ring of Vaseline was formed on the slide and this small "basin" was filled with amphipod saline and covered with small cover slips. By a careful movement of the cover slips the embryos can be arranged in a suitable position and arrested with a little pressure on the slip's top.

The fundamentals of 4D-microscopy are described by Schnabel et al. [38]. The image stacks were analyzed with the software SIMI°BioCell (SIMI, Germany). The data are illustrated as 3D-representations with color coded spheres. About 15 embryos were observed during the first row formation up to the row-like formation of the region E(0).

### In-vivo labeling

The cell labeling was done with an inverse microscope equipped with a micromanipulator (Leica DMIRB). The eggs in the relevant stages were put on microscopic slides under small cover slips that were equipped with plasticine feet at the corners. The eggs could be brought into the desired position by carefully shifting the cover slip. The eggs were held for the injection with soft pressure on the cover slip.

To get suitable needles for the injection, pipettes (Hilsberg, diameter 1.0 mm, thickness 0.2 mm) were pulled (KOPF Puller 720). After pulling the tips of the needles are closed and had to be open and sharpened with a horizontal grinder (Bachofer). The angle of the cutting edge varied between 20 and 30 degrees. The fluorescent marker was sucked into the injection-needle. DiI (Molecular Probes) was used as a vital marker. DiI is a lipophilic fluorescence-dye that binds to the cell membrane. This guarantees that the dye is exclusively restricted to the daughter cells. After a defined period of development the labeled eggs were observed with a fluorescence-microscope (Zeiss Axiophot1) using blue light or green light (strongest stimulation of DiI), and the results were documented with a digital camera (Nikon D1).

### CLSM and 3D-reconstruction

For fixation and documentation on the laser scanning microscope (Leica SP2) the embryos were dissected in PBS-buffered 4% formaldehyde-solution, counterstained with nuclear staining dye (Hoechst) and mounted in DABCO-Glycerol (25 mg DABCO (1,4 diazabicyclol-2,2,2-octane, Merck) in 1 ml PBS to 9 ml glycerol), which is an anti-bleaching-detergent. The image stacks produced by the laser scanning microscope were analyzed with the software Imaris 5.0.1 (Bitplane AG). The 3D-reconstruction of the counter staining (Hoechst) and the clones of the in-vivo labeled cell have the advantage of very high resolution with respect to morphological data. The feature "Volume" in the program module "Surpass" created a three dimensional object, which can be magnified and moved in all directions. For better visualization of the objects, movies (AVI-files) were created in the program module "Animation".

### Nomenclature

For the following description we adopt the common nomenclature for malacostracan crustacean cell lineages which was modified for amphipods by Scholtz [36]. In addition we introduce a nomenclature for the ectoderm row E(0). The anterior row of E(0) is named E(0)a and the posterior row E(0)p. If after the subsequent divisions a cell lies anterior it is again labeled with an "a". The posterior sister cell is labeled correspondingly with "p". As in more posterior rows the cells in positions to the midline (these cells and their early descendants are designated as columns) are numbered consecutively from the middle towards lateral (e.g. E(0)p1).

## Results

### The early cell lineage of the mandibular region (E(0))

**Figure 3:**
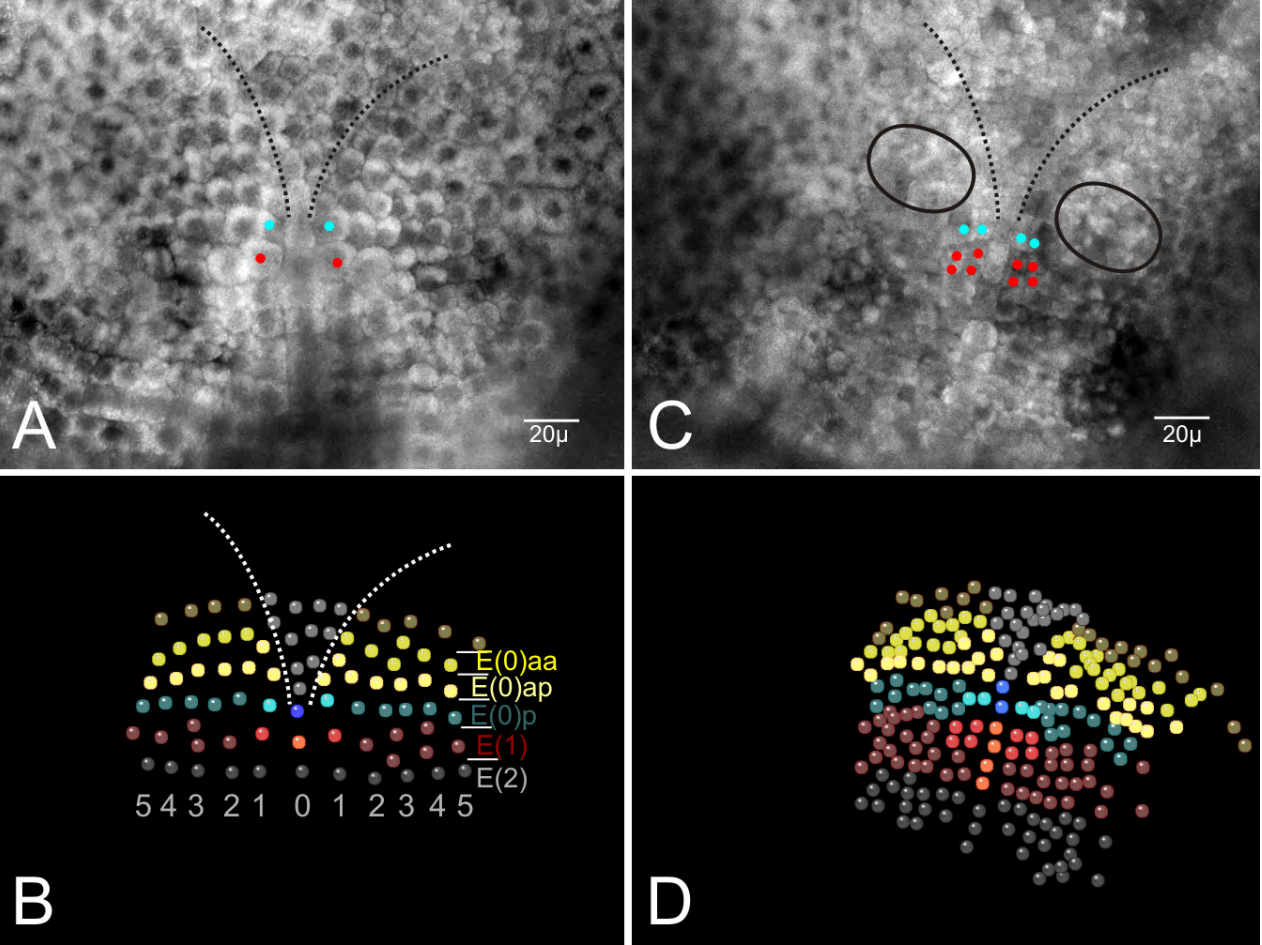
Using 4D-microscopy for detecting mandibular cell division pattern. (**A**) One level of the 4D-microscopy image stack from the beginning grid formation. The first transverse rows are already established. The light blue dots mark the cells E(1)1 in row E(1). The red dots above mark the cells E(0)p1. The dotted black lines mark the region of the prospective left and right head lobes. (**B**) Created SIMI°BioCell-model of cell arrangement of the ventral ectoderm of the embryo shown in figure **A**. The cells which are interesting to trace are color coded. Each genealogical region (row) has a distinct color (red: row E(1), blue: row E(0)p, light yellow: row E(0)ap, dark yellow: E(0)aa). The Arabic numbers sign out the different columns. (**C**) The embryo shown in **A **further developed (about 24 h). One level of the 4D-microscopy image stack from the ending of the time lapse recording. Because of the beginning morphogenesis of the limb buds it is impossible to reconstruct the cellular arrangement only by one slide. Most of the cells are not in focus. As in **A **the daughter cells of E(1)1 are marked with light blue dots, and the daughter cells of E(0)p1 with red dots. The circles indicate the early mandibular buds. (**D**) Created SIMI°BioCell-model of cell arrangement of the area shown in figure **C**. The traced cells are color coded as in **B**. Note that the genealogical unit E(1) (dark red) has its typical grid pattern. Within the unit E(2) (grey) it was not possible to trace all cells, some of them missing in this model.

After gastrulation, the blastopore is closed. The germ disc consists of about 250 cells (Fig. 2A). The descendants of the macromeres B (left) and D (right) form the anterior parts of the germ disc by migration towards the middle (for details see Wolff and Scholtz [39]). At this time there is no regular cellular pattern in the ventral ectoderm. The first recognizable transverse ectoderm row occurs in a germ disc with about 400 cells (Fig. 2B). This row marks the border between the naupliar (head lobes, the segments of the first and second antennae and of the mandibles) and the post-naupliar (the segment of the first maxillae up to the telson anlage) regions of the developing embryo. This first recognizable cell row forms the genealogical unit E(1) which provides most of the material for the segment of the first maxilla (Fig. 2C). The cells anterior to E(1) show a typical spatial configuration. According to Scholtz [36] we named this region E(0), which forms part of the prospective mandibular segment (Fig. 2C). The cells of E(0) are somewhat smaller and rounder than the more posterior cells. They are arranged in two rows of at least 6 cells on either body half (Fig. 2C, 3A–B). The anterior row is designated as E(0)a and the posterior row as E(0)p (Fig. 3A,B). It is not clear whether these two rows originate from one transverse ectoderm row by longitudinally oriented cell divisions. A distinct unpaired column of midline cells as is typical for the post-naupliar region (Fig. 2E) does not exist in the naupliar part of the germ band. In the median area of the posterior naupliar region E(0) we found about 10–15 smaller cells in a V-shaped arrangement (Fig. 3A,B).

**Figure 4:**
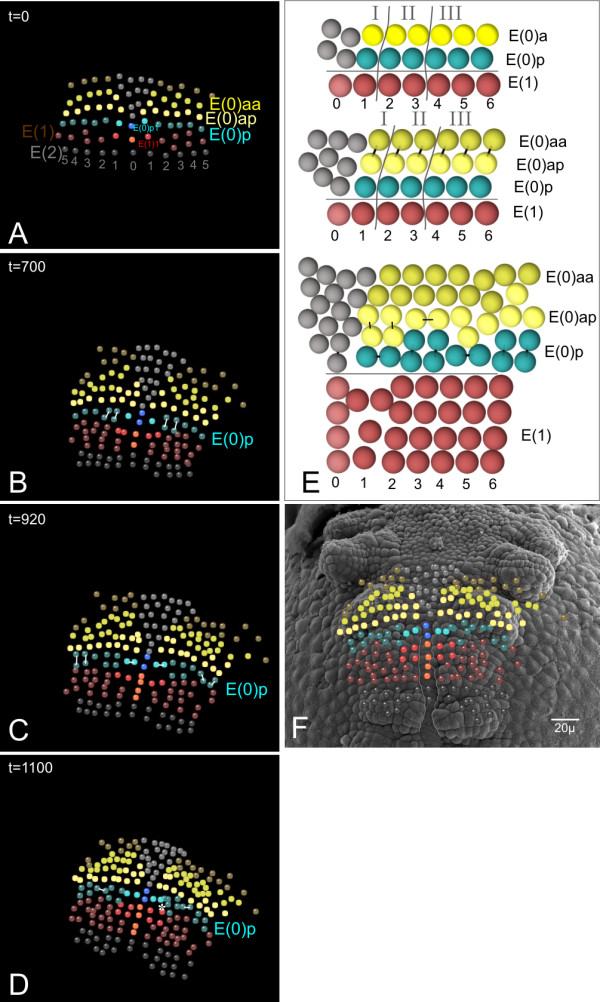
Cell division pattern of the mandibular region E(0) on the basis of a 4D-analysis. (**A**-**D**) Sequence of the early embryonic development with focus on cell lineage in region E(0) created with SIMI°BioCell. Each genealogical row is color coded. The strict cell divisions are marked by white lines. (**E**) Scheme of the cell division pattern in the region E(0). The color code and the labeling are the same as in the SIMI°BioCell-reconstruction. The median (midline) region is colored gray. Top: Time point of first visible row arrangement (E(1)) in post-naupliar germ band. At this time the prospective mandibular segment consists of two cell rows (E(0)a and E(0)p) of about 10–12 cells each. Roman numerals indicate the different areas in the mandibular segment. The horizontal line between E(0)p and E(1) marks the genealogical border between these rows, the segmental boundary between the mandibular segment and the segment of the first maxilla, and the border between the naupliar and the post-naupliar regions of the germ band. (**F**) Photo composition of mandibular cell lineage pattern and SEM picture found in *Orchestia*. The embryo of the SEM picture is about one cell division cycle in advance. However, at this developmental stage the naupliar segments (antenna 1, antenna 2 and mandible) have small appendage buds, whereas the adjacent posterior region shows only its segmental appearance. The substantial participation of row E(0)aa, E(0)ap and E(0)p in the outgrowth of the mandibular bud is visible.

At about this stage, cells of the anterior row E(0)a divide more or less synchronously (mitotic wave) in a longitudinal direction. The products are the anterior row E(0)aa and the posterior row E(0)ap (Fig. 3A,B). The following division pattern of cells in row E(0)aa is not clear in detail but the cells divide symmetrically in each body half (see supplemental material). In row E(0)ap a detailed pattern could not completely be described but some features of the more medially lying cells of this row could be traced. The cells E(0)ap1 und E(0)ap2 are the first to divide in a longitudinal direction whereas E(0)ap3 divides a bit later in a horizontal direction (Fig. 4E). The cell E(0)ap4 shows a delayed division. In relation to the division cycles in row E(0)a, the cells of E(0)p are delayed in their division.

During the second mitotic wave in row E(0)aa, E(0)p2 is the first cell that divides in row E(0)p. It divides in a longitudinal direction. More or less at the same time the midline cell E(0)p0 divides also in a longitudinal direction. Subsequently the cells E(0)p3, E(0)p5, and E(0)p6 divide likewise in a longitudinal direction E(0)p2 (Fig. 4B,C). At the same time the relatively large cell E(0)p1 divides in a horizontal direction (Fig. 4C). At last, the cell E(0)p4 undergoes a division, again in a horizontal direction (Fig. 4D). In this developmental stage small buds of the naupliar appendages (first and second antennae, mandible) become visible (Fig. 4F). This invariant cleavage pattern in the prospective mandibular region eventually produces a reproducible arrangement of cells in the posterior region of E(0)(Fig. 4E).

This is an important pre-condition for our detailed lineage study of the mandibular segment. Unfortunately, we could not reconstruct the complete lineage pattern of the more anterior lying region E(0)a.

### The clonal composition of the mandibular segment and its appendages

Identified cells of the transverse ectoderm rows around the boundary between the naupliar and post-naupliar regions were labeled in-vivo during the early germ band stage. The identification of cells is the prerequisite for the analysis of further cell fates and the clonal composition of morphological structures. Since a single cell approach was not feasible throughout, we divided the region E(0) from median to lateral into three sections. The median area, area I, begins lateral to the midline and ends before E(0)p2. The area II starts from E(0)p2 and comprises cell E(0)p3. The most lateral area III reaches from E(0)p4 to the end of the visible row formation (Fig. 4E). The successful in-vivo markings could be assigned to the corresponding areas.

### The midline-region of E(0)

The median region of the mandibular segment does not consist of an unpaired column as is known for the post-naupliar ectoderm (the so-called midline, see [40,41]). From the onset, about 10 smaller cells are more sunken into the yolk than are the surrounding symmetrically arranged cells. Thereby, the median cells form a typical V-shaped region (Fig. 3A). During ongoing development a clear separation (in comparison with surrounding lateral cells) is recognizable (Figs. 3C, 4F). The surface of this median region loses its cellular character and has a smooth appearance.

### The fate of area I

In relation to the more lateral adjoining cells (area II and III) the cells of area I of E(0) are retarded in cell division during segment formation. They form only a small portion (10–15 cells) of the early mandibular segment, and have their origin in the first column of region E(0) – E(0)a1 and E(0)p1.

In vivo labeling of cell E(0)a1 shows that its descendants form the medio-anterior part of the developing humps of the paragnaths. In addition, a median part of the mandibular sternite is formed by E(0)a1-descendants.

**Figure 5:**
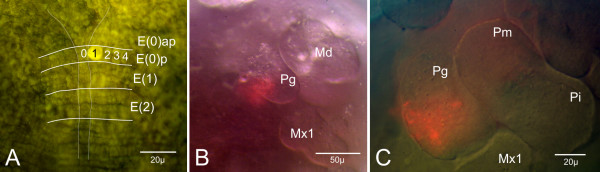
In-vivo labeling of area I in region E(0). (**A**) Double exposure of an in-vivo labeled embryo. The ventral ectodermal rows are marked with white lines and labeled with nomenclature. The fluorescent dye is completely diffused in the membrane of cell E(0)p1(of the left body half). (**B**) Same embryo as in **A **further developed. Ventral view of head region. The descendants of the marked cell form postero-median parts of the sternite (blurry because not in focus) and of the mandible (Md). (**C**) Embryo as in **B **further developed. Ventral view of the left mandibular segment. The mandible consists of two lobes, the inner pars molaris (Pm) and the outer pars incisivus (Pi). Few E(0)p1-clones form postero-median parts of the paragnath hump (Pg). The sternal clones seen in **B **are out of focus.

The descendants of E(0)p1 form postero-median parts of the paragnaths and adjacent areas of the sternite (Fig. 5A–C, Additional file 1). A few clones of E(0)p1 are found within the neuro-ectoderm in the posterior region of the mandibular segment and form nervous structures. Finally, after further development the ectodermal descendants are involved in the formation of postero-median parts of the paragnaths.

In general, area I forms the median mandibular sternite and median parts of the paragnaths. The clonal composition of a developed paragnath and developed parts of the sternite reflects the early a/p-arrangement of the rows.

### The fate of area II

**Figure 6:**
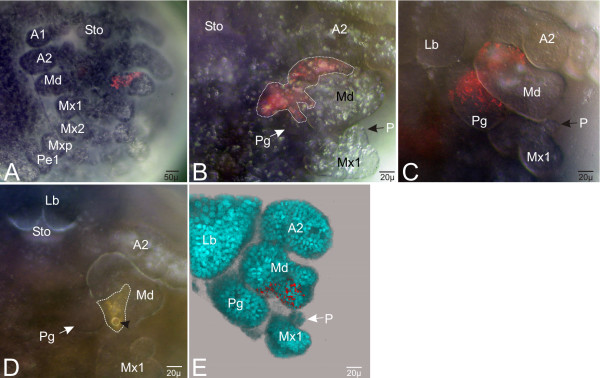
In-vivo labeling of area II in region E(0). The images are in ventral view and double exposure. (**A-C**) Labeling of cell E(0)a2 of the left body half. (**A**)Anterior embryonic region which at this time lacks visible paragnath buds. The descendants of E(0)a2 form anterior parts of the mandible bud (Md) as well as parts of the developing sternite. (**B**) Left gnathal region of the embryo as in **A **further developed. The small palp (P) of the maxilla 1 (Mx1) is now formed. There are two distinct clone domains. One forms anterior part of the small paragnath bud (Pg), the other forms anterior parts of the developing mandible (Md). (**C**) Left mandibular region of the embryo as in **B **after further development. The paragnaths grow out and the mandible differentiates into two lobes. The clonal composition of the descendants of E(0)a2 is more or less the same as in (**E**). (**D**-**E**) Labeling of cell E(0)p3 of the left body half. (**D**) Ventral view of the mouth region. The descendants of the marked cell form posterior parts of the mandibular bud (Md), but they do not form parts of the anlage of the paragnath (Pg). The arrow head shows the oil drop of the marking. (**E**) Same embryo as in **D **slightly further developed. Imaris-reconstruction of the left gnathal region. The blue spots represent the counter-stained (Hoechst) nuclei. The descendants of E(0)p3 are involved in the formation of posterior parts of the mandible, which now consists of two well developed lobes. For detail see Additional file 2.

The lateral adjoining area of area I is area II, which comprises the descendants of column 2 and 3. It forms lateral parts of the paragnaths and median parts of the mandible. Descendants of column 2 are found in more lateral parts of the paragnath and in antero-median parts of the mandible (Fig. 6A–C, Additional file 2). Interestingly, these median parts of the early mandibular bud are later differentiated into the molar process.

The cells of column 3 give rise to median parts of the mandible (Fig. 6D–E, Additional file 2). As in area I, the descendants of the anterior row E(0)a form more anterior parts and according to that the descendants of row E(0)p proliferate more posterior parts. As well, some cells of the sternite have their origin in cells of area II.

In general, area II is responsible for the formation of lateral parts of the paragnaths and more median parts of the mandibles.

### The fate of area III

**Figure 7:**
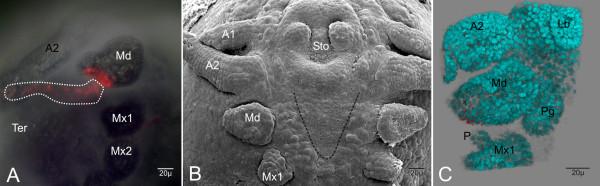
In-vivo labeling and clonal composition of area III using the example of cell E(0)p4. (**A**) Ventral view (double exposure) of the right head region. The lateral part of the mandible is formed by descendants of E(0)p4 as well as adjacent parts of the tergite (white dotted line). Postero-lateral parts of the mandible and parts of the adjacent tergite (Ter) is formed by descendant of cell E(0)p4. (**B**) SEM-picture of an embryo in an almost the same stage as the embryo shown in **A**. Only the triangular shaped mandible (Md) and not the anlagen of the paragnaths are developed. The dotted line indicates the slightly deeper medial cells of region E(0). (**C**) The same embryo as in (**A**) further developed. Imaris-reconstruction of the left mouth region of the fixed embryo. The blue spots are the counter-stained (Hoechst) nuclei. The E(0)p4-clones form the outer (lateral) tip of the young mandible. The rest of the clones (visible in **A**) were cut off. For detail see Additional file 3. (antenna 1 = A1; antenna 2 = A2; labrum = lb; maxilla 1 = Mx1; maxilla 2 = Mx2; maxilliped = Mxp; palpus = P; stomodaeum = Sto)

Area III lies laterally adjacent to area II and comprises columns 4 and 5 of region E(0). They form lateral parts of the mandible and neighboring parts of the tergite. That is why the cell E(0)p4 produces cell material for the outer (postero-lateral) part of the mandible and lateral bordering tergite (Fig. 7A–C, Additional file 3). In contrast, descendants of row E(0)a in area III form more anterior parts of the outer (antero-lateral) mandible and parts of the adjacent tergite. Remarkably, the lateral portion of the mandibles differentiates by cell proliferation into the prospective pars incisiva.

Summarizing for area III, column 4 forms lateral parts of the mandibles and adjacent tergites and column 5 is not involved in the development of mandibles or paragnaths.

### Surrounding areas of E(0)

#### anterior

The fate of the cells anterior to the region of E(0) has not yet been resolved in detail. Furthermore, it is not clear if there is a stereotypic cell division pattern at all forming the anterior embryonic head. Some labeling reveals that median cells of E(0) form part of the stomodaeum and more lateral cells form part of the second antennae.

#### posterior

Beginning with row E(1) the typical grid-like pattern of the post-naupliar germ band of *Orchestia *is established. The cell division pattern of E(1) differs somewhat from that of the more posterior rows (compare Figs. 2D and 4E) (for details see [36]). The 4-D analysis in row E(1) shows that its descendant cells are not involved in the formation of the mandibular segment (Fig. 4E) Hence, in contrast to the post-naupliar segments, in the mandibular segment the posterior genealogical boundary corresponds with the segment border.

## Discussion and conclusion

### A stereotyped cell division pattern is found at the posterior border of the mandibular segment

Like all malacostracan crustaceans studied in this respect (for a recent review see Dohle et al. [42]), amphipods show a stereotyped cell divisions pattern in the post-naupliar region during growth, differentiation and segmentation of the germ band [36,43,44]. In contrast to this, the naupliar region does not exhibit an obvious stereotyped cell division pattern [37,42]. Only Scholtz [36] suggested that there might be a certain regularity in the divisions and arrangements of the posterior cells of the developing mandibular segment in the amphipod *Gammarus pulex *but with the methods then at hand the details were not resolvable With the technique of 4D-microscopy we have been able to provide the first evidence for an invariant cell division pattern in the naupliar region of another amphipod species, the freshwater beach hopper *Orchestia cavimana*. At least in the posterior part of the region of E(0) we recognized a relatively strict cell division pattern. This pattern has only superficial similarities to the post-naupliar cell division pattern of malacostracan crustaceans but it is not as elaborated in terms of timing of mitoses, cell size, and the spatial arrangement of the resulting cells. In E(0) the sequence of the individual divisions is not as strict and the direction of the mitotic spindles is more or less longitudinally oriented. It appears that the posterior border of the mandibular segment forms some kind of transition between the more irregular divisions and cell arrangements of the anterior naupliar region and the highly complex stereotyped post-naupliar patterns.

It is not clear whether our findings of a regular pattern in the posterior part of the mandibular segment holds true for other malacostracans as well, although some data from the isopod *Porcellio scaber *hint to that possibility [45]. However, the cellular events in the corresponding region in *Porcellio *are much more irregular when compared with those in *Orchestia*. It has been even shown for *Porcellio *that some cells of the most anterior row of the post-naupliar segments can migrate into the posterior area of the mandible segment [45], a phenomenon that does not occur in *Orchestia*.

Interestingly enough, our results reveal that the posterior segmental boundary of the mandibular segment corresponds to the genealogical border between rows E(0) and E(1), i.e. E(1) does not contribute to the posterior part of the mandibular segment. This stands in contrast to all more posterior post-naupliar segmental boundaries which are formed within the descendants of one ectoderm row and thus do not match the genealogical borders (see Fig. 2), [42]. Row E(1) forms a kind of transition between these segmentation modes because its posterior region follows the typical post-naupliar pattern in that it contributes to the anterior portion of the segment of the first maxillae whose posterior part is formed by anterior descendants of the next adjacent row E(2) [36,37]. These differences indicate that the parasegmental organization (i.e. a frame-shift between the early metameric anlagen and the resulting morphological segments) of the post-naupliar germ band (see [42]) is not found in the naupliar region with a transition in the first maxillary segment.

### Cell lineage data and clonal analyses reveal that paragnaths are not limbs but outgrowths of the sternal region of the mandibular segment

Based on our knowledge of the cell division pattern of the early developing mandibular region (see above) we were able to look at its morphogenesis at a very high level of resolution. By means of single cell labeling with the fluorescent dye DiI we were able to reconstruct and analyze the clonal composition of the mandibular region from the beginning of ectodermal proliferation up to the differentiation of the mouthparts. The cell labeling reveals that the paragnaths have their origin in the area I and area II which comprises columns 1 to 3 of region E(0). The mandibles originate from cells of the areas II and III (columns 2 to 4). Areas I and II contribute also to the sternal region and the mandibular ganglia whereas area III forms parts of the tergites as well. In more posterior segments, columns 1 and 2 mainly contribute to the formation of segmental ganglia and probably sternites, and columns 3 to 5 mainly give rise to limbs [36,46]. Hence, when compared with clonal composition of the post-naupliar segments it is evident that the columns that form the ganglia and sternites in these segments correspond to those that give rise to the paragnaths in the mandibular segment. In addition, the mandibular buds are formed in a comparable position to the other limbs. This clearly reveals that paragnaths of *Orchestia *are processes of the mandibular sternal region.

Further evidence for the claim that crustacean paragnaths belong to the mandibular segment is based on gene expression data. For instance, the segment polarity gene *engrailed *is expressed in a regular stripe in the posterior region of the mandibular segment, as in all other segments, comprising cells that form the posterior part of the paragnaths in amphipods, isopods and decapods [14,18,37]. Moreover, the expression of the Hox-gene *Deformed (Dfd) *is mainly found in the mandible segment of hexapods [47,48], myriapods [49,50], and crustaceans [14,16], and in the latter case expression of *Dfd *comprises the buds of the paragnaths [14,16].

All these data reveal that paragnaths are part of the mandibular segment and that they are not limb derivatives as has been suggested by several authors. This confirms previous ideas on the origin and nature of paragnaths based on embryological and larval evidence [17]. With our clonal analysis we can definitely rule out the possibility that paragnaths indicate the existence of an additional head segment as has been claimed for example by Casanova [29], Chaudonneret [26], Denis [34], and Hansen [28]. Furthermore, the limb-bud like early appearance of the paragnaths in *Orchestia *and other crustaceans is no indication for a limb-related nature of these structures but is only a superficial similarity that does not represent a genealogical relation. Our results show the power of cell lineage and clonal analyses for inferences on the nature, origin and thus homology of morphological structures. With this kind of investigation morphological and gene expression data can be complemented.

In many cases the origin of the paragnaths during crustacean embryonic and larval development has either not been specified (e.g. Ref. [51-53]) or it has been suggested that paragnaths develop from mandibular and/or maxillary segments (e.g. Ref. [54]). A look at the corresponding figures in these articles with our results in mind reveals that it is possible in almost all examples to relate the paragnathal structures to the mandibular segment (e.g. Manton [51], plate 24, fig. 27; Manton [52], plate 25, fig. 23; Moeller [53], figs. 2, 7). Even the median lobe of the so-called lower lip of the raptorial cladoceran *Leptodora kindtii *might represent fused paragnaths [55]. Accordingly, we tentatively conclude that the paragnaths lobes are homologous throughout Crustacea. Stein et al. [56] suggest that a pair of paragnaths humps is an apomorphy for a crustacean subgroup comprising Eucrustacea and Phosphatocopina (Labrophora). However, these authors did not consider similar structures in myriapods and hexapods which indicate a much more widespread occurrence among euarthropods. (see next chapter).

### Are the crustacean paragnaths homologous to the superlinguae in hexapods and myriapods?

There are several reports of post-oral paired bud-like anlagen in myriapods (e.g. Chilopoda: Heymons [57], Progoneata: Tiegs [19]) and Hexapoda (e.g. Ref. [24,25,58-60]). In dicondylian hexapods the situation is somewhat ambiguous. Larink [61] and Scholl [62] report for *Lepisma *and *Carausius *an undivided early hypopharynx anlage whereas Rohrschneider [24] for *Periplaneta *and Ibrahim [63] for *Tachycines *describe two separated buds. Whether these are factual differences is not clear. However, the presence of paired buds in a number of pterygotes as well as collembolans, diplurans, and archaeognathans allows the conclusion that these paired buds were present in the hexapod stem species. Nevertheless, all these buds have in common that they originate from the sternum of the mandible segment directly ventral to the mandibular ganglion anlagen and between the mandibular limb buds, after these have formed. This very much resembles the early stages of crustacean paragnaths (see above, [14,17,18,51,52]). These sternal buds of the mandibular segment give either rise to the superlinguae, paired lateral lobes of the hypopharynx, in Symphyla [19], Collembola [58,59], Diplura, Archaeognatha [25], or to (another part of) the hypopharynx [22,24,57] when superlinguae are not present as for instance is the case in many Pterygota and all Chilopoda [22,64]. However, the contribution of these mandibular sternal buds to the hypopharynx body (linguae) is interpreted controversially. The hypopharynx of Hexapoda and Progoneata is thought to be a composite structure formed by protrusions of the sternites of different numbers of gnathal segments (e.g. Ref. [19,22,25,61,62]). In contrast to this, Haget [65] and Wada [66] suggest, based on their experimental teratological studies that the entire hypopharynx originates from the mandibular sternites. According to Heymons [57], this is also the case in Chilopoda. The situation is further complicated by the fact that buds appearing in the intercalary segment of hexapods are called "hypopharyngeal lobes" (Hypopharynxhöcker) and have been suggested to form (part of) the hypopharynx, an obvious misinterpretation (see Ref. [23,63,67]). Reading the numerous articles dealing with this problem it is evident that investigations focusing on the differentiation of the hypopharynx in myriapods and hexapods using modern approaches are badly needed to solve this issue.

Authors such as Crampton [27], Snodgrass [68], and Bitsch and Bitsch [5] suggest that the paragnaths and the superlinguae are homologous. However, based on our data and what we find in the above discussed literature about the development of the hexapod and myriapod hypopharyngeal complex, we think that the conclusion of these authors is simplifying the matters because paragnaths cannot be homologous to superlinguae alone if the major part of the hypopharynx of myriapods and hexapods originates from the mandibular sternites as well. Accordingly, we modify the homology statement concerning crustacean paragnaths and the myriapod/hexapod superlinguae/hypopharynx by suggesting that the *early anlagen *of these structures are homologous, taking into account that the homology of early developmental stages does not necessarily mean that more advanced stages are also homologous [69]. Since a comparable structure is absent in the corresponding segment of Chelicerata and Onychophora (first walking leg, see Ref. [1,2,70-72]) it is likely that a pair of sternal buds in the mandibular segment is a shared apomorphy of Crustacea, Myriapoda, and Hexapoda. Accordingly these structures provide further support for the Mandibulata hypothesis (see also Ref. [2,73-75]) which has been disputed based on molecular data (e.g. Ref. [76-78]).

## Supplementary Material

Additional file 1 Example of the clonal composition of area I on hand cell E(0)p1.Detail of the left mouth region (see Fig. 5). For better orientation the mandible and labrum are marked. The descendants of E(0)p1 form postero-median parts of the outgrowing paragnath. Additionally adjacent parts of the sternum are formed by these cells. You have to go with the mouse cursor into the frame. By pressing the left mouse button you can move the reconstruction in any position. Click here for file

Additional file 2 Example of the clonal composition of area II on hand cell E(0)p3. Detail of the left mouth region (see Fig. 6). For better orientation the mandible and labrum are marked. The descendants of the marked cell form postero-median parts of the mandible. Some clones are outside the mandible. You have to go with the mouse cursor into the frame. By pressing the left mouse button you can move the reconstruction in any position. Click here for file

Additional file 3 Example of the clonal composition of area III on hand cell E(0)p4. Detail of the right mouth region (see Fig. 7). For better orientation the mandible and labrum are marked. The clones of the marked cell form lateral parts of the mandible and adjacent parts of the tergite. These cells were cut off during dissection, check the Fig. 7A. You have to go with the mouse cursor into the frame. By pressing the left mouse button you can move the reconstruction in any position. Click here for file
